# Influence of the combination of SGLT2 inhibitors and GLP-1 receptor agonists on eGFR decline in type 2 diabetes: post-hoc analysis of RECAP study

**DOI:** 10.3389/fphar.2024.1358573

**Published:** 2024-03-27

**Authors:** Yoshimi Muta, Kazuo Kobayashi, Masao Toyoda, Atsuhito Tone, Daisuke Suzuki, Daisuke Tsuriya, Hideo Machimura, Hidetoshi Shimura, Hiroshi Takeda, Hisashi Yokomizo, Kei Takeshita, Keiichi Chin, Keizo Kanasaki, Kouichi Tamura, Masaaki Miyauchi, Masuo Saburi, Miwa Morita, Miwako Yomota, Moritsugu Kimura, Nobuo Hatori, Shinichi Nakajima, Shun Ito, Shunichiro Tsukamoto, Takashi Murata, Takaya Matsushita, Takayuki Furuki, Takuya Hashimoto, Tomoya Umezono, Yuichi Takashi, Daiji Kawanami

**Affiliations:** ^1^ Department of Endocrinology and Diabetes, Fukuoka University School of Medicine, Fukuoka, Japan; ^2^ Department of Medical Science and Cardiorenal Medicine, Yokohama City University Graduate School of Medicine, Kanagawa, Japan; ^3^ Division of Nephrology, Endocrinology and Metabolism, Department of Internal Medicine, Tokai University School of Medicine, Kanagawa, Japan; ^4^ Department of Internal Medicine, Diabetes Center, Okayama Saiseikai General Hospital, Okayama, Japan; ^5^ Suzuki Diabetes Clinic, Kanagawa, Japan; ^6^ Division of Endocrinology and Metabolism, 2nd Department of Internal Medicine, Hamamatsu University School of Medicine, Shizuoka, Japan; ^7^ Machimura Internal Medicine Clinic, Kanagawa, Japan; ^8^ Shimura Clinic, Kanagawa, Japan; ^9^ Takeda Clinic, Kanagawa, Japan; ^10^ Hakuai Clinic, Kanagawa, Japan; ^11^ Department of Internal Medicine 1, Endocrinology and Metabolism, Shimane University Faculty of Medicine, Shimane, Japan; ^12^ Miyauchi Diabetes Clinic, Kanagawa, Japan; ^13^ Department of Diabetology, Endocrinology and Metabolism, Tokyo Medical University Hachioji Medical Center, Tokyo, Japan; ^14^ Kobayashi Hospital, Kanagawa, Japan; ^15^ Sagami Junkanki Clinic, Kanagawa, Japan; ^16^ Department of Internal Medicine, Sagamihara Red Cross Hospital, Kanagawa, Japan; ^17^ Department of Clinical Nutrition, National Hospital Organization Kyoto Medical Center, Kyoto, Japan; ^18^ Diabetes Center, National Hospital Organization Kyoto Medical Center, Kyoto, Japan; ^19^ Hadano Station South Gate Clinic, Kanagawa, Japan; ^20^ Umezono Internal Medicine Clinic, Kanagawa, Japan

**Keywords:** sodium-glucose cotransporter 2 inhibitors, glucagon-like peptide 1 receptor agonists, renal outcome, combination therapy, preceding drug, diabetic kidney disease

## Abstract

Accumulating evidence has demonstrated that both SGLT2 inhibitors (SGLT2i) and GLP-1 receptor agonists (GLP1Ra) have protective effects in patients with diabetic kidney disease. Combination therapy with SGLT2i and GLP1Ra is commonly used in patients with type 2 diabetes (T2D). We previously reported that in combination therapy of SGLT2i and GLP1Ra, the effect on the renal composite outcome did not differ according to the preceding drug. However, it remains unclear how the initiation of combination therapy is associated with the renal function depending on the preceding drug. In this *post hoc* analysis, we analyzed a total of 643 T2D patients (GLP1Ra-preceding group, *n* = 331; SGLT2i-preceding group, *n* = 312) and investigated the differences in annual eGFR decline. Multiple imputation and propensity score matching were performed to compare the annual eGFR decline. The reduction in annual eGFR decline in the SGLT2i-preceding group (pre: −3.5 ± 9.4 mL/min/1.73 m^2^/year, post: −0.4 ± 6.3 mL/min/1.73 m^2^/year, *p* < 0.001), was significantly smaller after the initiation of GLP1Ra, whereas the GLP1Ra-preceding group tended to slow the eGFR decline but not to a statistically significant extent (pre: −2.0 ± 10.9 mL/min/1.73 m^2^/year, post: −1.8 ± 5.4 mL/min/1.73 m^2^/year, *p* = 0.83) after the initiation of SGLT2i. After the addition of GLP1Ra to SGLT2i-treated patients, slower annual eGFR decline was observed. Our data raise the possibility that the renal benefits—especially annual eGFR decline—of combination therapy with SGLT2i and GLP1Ra may be affected by the preceding drug.

## 1 Introduction

Diabetic kidney disease (DKD) is the leading cause of end-stage kidney disease (ESKD) worldwide (Kim and Kim, 2022). Preventing the onset and progression of DKD is important for preventing ESKD and cardiovascular disease (CVD) (Jankowski et al., 2021). DKD is characterized by albuminuria and estimated glomerular filtration rate (eGFR) decline (Norris et al., 2018). However, recent studies have shown that the prevalence of impaired kidney function with normo-albuminuria is increasing among patients with type 2 diabetes (T2D) (Kume et al., 2019), and those individuals are at increased risk for ESKD and all-cause mortality (Yamamoto et al., 2022). These data suggest that the pathophysiology of DKD is complex.

SGLT2 inhibitors (SGLT2i) and GLP-1 receptor antagonists (GLP1Ra) are widely used for the treatment of T2D and have been shown to have potent glucose-lowering and weight loss effects. Accumulating evidence has demonstrated that SGLT2i and GLP1Ra both have beneficial effects on DKD.

Approximately 40% of T2D patients develop chronic kidney disease (CKD) (Norris et al., 2018). In Japan, 9.9% of patients with type 1 diabetes (T1D) and 15.3% of patients with T2D have a reduced kidney function (eGFR <60) (Shikata et al., 2020). Therefore, it is important to establish an efficient therapeutic strategy against DKD using currently available drugs such as SGLT2i and GLP1Ra.

The International Diabetes Practice Guidelines for CKD, Kidney Disease Improving Global Outcomes (KDIGO) 2022, SGLT2i and metformin are listed as first-line drug therapies for diabetes-associated CKD, and GLP1Ra is preferred as second-line drug therapy (Rossing et al., 2022). Although the renoprotective effects of SGLT2i and GLP1Ra have been reported, the efficacy of their combination therapy has not been fully investigated. Because of side effects, excessive hypoglycemic effects, and medical costs, the initiation of both drugs at the same time is not clinically practiced, and one drug is added after the initiation of treatment with the initial drug. It has not yet been established which drug should be administered first from the viewpoint of renal protection. To clarify this point, we performed the RECAP study (the renoprotective effects of combination treatment with SGLT2i and GLP1Ra in patients with T2D according to their preceding medication). However, in the SGLT2i and GLP1Ra combination therapy, the preceding drug did not affect the renal composite outcome (Kobayashi et al., 2023).

Recently, eGFR decline was implicated as a surrogate endpoint for ESKD (Grams et al., 2019). Therefore, we performed a *post hoc* analysis of the RECAP study to examine 1) whether the combination therapy of SGLT2i and GLP1Ra is favorable for eGFR decline and 2) how the initiation of combination therapy influences eGFR decline depending on the preceding drug. Evaluations were performed before and after combination therapy with SGLT2i and GLP1Ra in T2D patients.

## 2 Materials and methods

### 2.1 Study design

The design of the RECAP study is shown in Supplementary Figure S1. T2D patients who received both SGLT2i and GLP1Ra from April 2010 to December 2021 who had received their preceding medication for at least 6 months, who had received concomitant medication for at least 12 months, and for whom clinical data were available from baseline, the time of drug addition, and the final observation were eligible for inclusion. The following data were collected: sex, age, height, body weight (BW), systolic blood pressure (SBP), diastolic blood pressure (DBP), eGFR, glycated hemoglobin A_1c_ (HbA_1c_), urinalysis results (urine albumin-to-creatinine ratio (ACR) [mg/g Cr] or qualitative proteinuria), alanine aminotransferase (ALT) level, aspartate aminotransferase (AST) level, platelet count, and concomitant medications (including hypoglycemic drugs, antihypertensive drugs, and statins). eGFR was determined as follows: eGFR (mL/min/1.73 m^2^) = 194 × age^−0.287^ × serum creatinine^−1.094^ × (0.739 for women) (Matsuo et al., 2009). Qualitative proteinuria values were converted to albuminuria values using the following formula: predicted ACR = exp (5.2659 + 0.2934 × log (min (PCR/50, 1)) + 1.5643 × log (max(min(PCR/500, 1), 0.1)) + 1.1109 × log (max (PCR/500, 1))-0.0773×(if female) + 0.0797×(if diabetic) + 0.1265×(if hypertensive))) (Sumida et al., 2020). Patients with any of the following conditions were excluded from the study: T1D; chronic dialysis; severe liver dysfunction (e.g., liver cirrhosis or severe infection), terminal-stage malignancy, pregnancy, treatment discontinuation, and those who opted out during the course of the study.

The details of the study participants and the dataset used in this study are shown in Supplementary Figure S2. Based on the inclusion criteria, we collected data of 688 patients from 18 medical facilities. Because of the exclusion criteria, 45 patients were excluded and 643 patients (331 patients were previously treated with SGLT2i and later treated with GLP1Ra [SGLT2i-preceding group], and 312 patients were previously treated with GLP1Ra and later treated with SGLT2i [GLP1Ra-preceding group]) were analyzed in this study. Because some data were missing from 643 patients, we performed a multiple imputation (MI) and this dataset complemented by MI was used for the main statistical analysis in this study as full analysis set (FAS). In contrast, after excluding patients with any missing data, 418 patients (227 in the SGLT2i-preceding group, and 191 in the GLP1Ra group) remained. We used this dataset without missing data, that is called complete case analysis set (CCA), to conduct a sensitivity analysis.

As shown in Supplementary Figure S1, patients were first treated with either SGLT2i or GLP1Ra, after more than 6 months, another drug was added and the combination therapy of SGLT2i and GLP1Ra was administered. Therefore, we collected data from three points; at baseline, at the time of drug addition, and at the final observation as shown in Supplementary Figure S1. The median (range) period from baseline to the time of drug addition (monotherapy period) was 23 months (6–114 months). The median (range) period from drug addition to the final observation (combination therapy time) was 31 months (12–85 months). The median (range) period from baseline to the final observation (total observation period) was 59 months (19–134 months) in the total study participants.

The present study was approved by the Institutional Review Board for Clinical Research of Tokai University, Japan on 6 December 2021.

### 2.2 Office BP measurement

The BP was measured as previously described (Kobayashi et al., 2019). Office BP measurements were performed at each institution using their validated cuff oscillometric devices. According to the JSH 2014 guidelines (Shimamoto et al., 2014), office BP was measured in a quiet environment after resting for a few minutes in a seated position on a chair with legs uncrossed. When two consecutive measurements were taken 1–2 min apart, the average value was defined as the office BP.

### 2.3 Outcomes

The annual eGFR decline (annual ΔeGFR) during monotherapy (SGLT2i or GLP1Ra) and after combination therapy was evaluated in the SGLT2i-preceding and GLP1Ra-preceding groups. We also evaluated the change in the logarithmic value of ACR (LnACR) in this *post hoc* analysis.

### 2.4 Statistical method

#### 2.4.1 Missing value analysis

MI was performed to account for missing values (Rubin, 1987). MI is used to replace missing values with other plausible values by creating multiple filling-in patterns to avoid bias caused by missing data. This is recognized as an alternative approach to the analysis of incomplete data (Rubin and Schenker, 1991). In the RECAP study, each missing value was replaced with a set of substituted plausible values by creating 100 complete datasets using MI with the chained equations method (Hershberger and Fisher, 2003; Enders, 2010; Aloisio et al., 2014). For imputation, 100 complete datasets were created using the following covariates: age, sex, height, BW, SBP, DBP, HbA_1c_, eGFR, LnACR, types of SGLT2i and GLP1Ra, use of concomitant medications (hypoglycemic drugs, antihypertensive drugs, and statins, and period of treatment with either or both SGLT2i or GLP1Ra). The clinical data at baseline, at the time of drug addition, and at the final observation that were associated with the outcome were used for MI (Supplementary Figure S2) (Furukawa et al., 2017).

#### 2.4.2 Statistical analysis for the description of data and comparison

Normally distributed data are presented as the mean ± standard deviation (SD). Data that showed a skewed distribution were reported as the median [25th percentile, 75th percentile]. For parametric variables, comparisons of clinical characteristics between the two groups were performed using an unpaired *t*-test, The chi-square test was used for nonparametric variables and for categorical data, while a general linear mixed model (GLMM) with Bonferroni correction was used to compare the clinical findings between the three points (at baseline, at the time of drug addition, and at the final observation) as described previously.

Adjusted eGFR decline was calculated by a multiple linear regression analysis using eGFR at baseline, sex, age, BW, BMI, SBP, DBP, HbA1c, and treatment periods in the CCA set.

Multiple regression analysis was performed to identify the independent factors related to change in eGFR decline before and after the combination treatment of SGLT2i and GLP1Ra. A stepwise method was performed using covariates as follows; sex, the duration of T2D, preceding drug, periods of the combination treatment, age, BMI, HbA1c, MAP, eGFR, LnACR at the time of drug addition, changes in BMI, HbA1c, MAP, and LnACR from at the time of drug addition to at the final observation. All statistical analyses were performed using IBM SPSS Statistics 28.0 (IBM Inc., Armonk, NY, USA). Statistical significance was set at *p* < 0.05.

#### 2.4.3 Propensity score matching

Differences were observed in the baseline characteristics of the SGLT2i-preceding and GLP1Ra-preceding groups. Adjustments were needed for comparison between the groups. Propensity score (PS) matching was conducted to compare the annual eGFR decline and change in albuminuria.

In each dataset built by MI, the PS for the SGLT2i-preceding group was calculated by a logistic analysis that included the following covariates: sex, age, height, BW, body mass index (BMI), SBP, DBP, HbA_1c_, eGFR, LnACR at baseline, history of T2D, concomitant medications at baseline (hypoglycemic drugs, antihypertensive drugs, and statins), and treatment periods for monotherapy and combination therapy. Because individual PSs were calculated using datasets built by MI, the average PS was used as the representative value. PS matching was performed using representative PS values with the following algorithm: 1:1 nearest neighbor match (caliper value = 0.047, calculated as 0.2 × the SD of PS (Austin, 2011)) with no replacement. In the PS-matching model, the paired *t*-test was used for parametric variables, and the McNemar test was used for categorical data.

## 3 Results

### 3.1 Baseline characteristics

The clinical characteristics at baseline of FAS with MI (*n* = 643) before and after PS matching in the RECAP study are shown in Table 1. Before PS matching, the SGLT2i-preceding and GLP1Ra-preceding groups showed significant differences in the history of T2D >10 years (76% vs. 85%, *p* = 0.006), SBP (mmHg) (135.4 ± 18.9 vs. 132.0 ± 18.4, *p* = 0.02), DBP (mmHg) (78.7 ± 13.6 vs. 76.6 ± 12.3, *p* = 0.04), period of monotherapy (month) (23.9 ± 14.0 vs. 31.8 ± 23.1, *p* < 0.001), period of combination therapy (month) (28.5 ± 13.5 vs. 38.8 ± 18.6, *p* < 0.001), total study period (month) (52.4 ± 15.7 vs. 70.6 ± 27.0, *p* < 0.001), use of metformin (61% vs. 51%, *p* = 0.01) and pioglitazone (16% vs. 11%, *p* = 0.03) at baseline. In the PS-matching model, the range of standardized differences in covariates was 0.0–0.12. Therefore, the PS-matching model was considered to be well-balanced between the groups.

**TABLE 1:** Clinical characteristics at baseline.

	Unadjusted model			PS-matching model		
	GLP1Ra-preceding group, *n* = 331	SGLT2i-preceding group, *n* = 312	*p*-value	GLP1Ra-preceding group, *n* = 203	SGLT2i-preceding group, *n* = 203	Standardized difference
Age (years)	55.7 ± 13.5	56.5 ± 12.7	0.10	57.1 ± 13.6	57.0 ± 13.2	0.007
Sex (female [%])	152 (46%)	130 (42%)	0.27*	89 (44%)	87 (43%)	0.02
History of type 2 diabetes >10 years (%)	281 (85%)	237 (76%)	0.006*	165 (81%)	159 (78%)	0.07
BW (kg)	79.5 ± 20.1	79.4 ± 18.1	0.95	78.7 ± 18.5	78.8 ± 17.0	0.006
BMI	29.8 ± 6.3	29.5 ± 5.6	0.51	29.4 ± 5.5	29.2 ± 5.3	0.04
SBP (mmHg)	132.0 ± 18.4	135.4 ± 18.9	0.02	133.1 ± 19.1	134.7 ± 19.4	0.08
DBP (mmHg)	76.6 ± 12.3	78.7 ± 13.6	0.04	76.7 ± 12.4	78.2 ± 13.5	0.12
HbA_1c_ (mmol/mol [%])	73.6 ± 18.6 (8.9 ± 1.7)	71.0 ± 17.3 (8.6 ± 1.6)	0.07	72.8 ± 17.8 (8.7 ± 11.6)	71.9 ± 18.2 (8.7 ± 1.7)	0.05
eGFR (mL/min/1.73 m^2^)	78.8 ± 28.7	78.2 ± 26.0	0.79	76.6 ± 26.7	77.7 ± 26.9	0.04
LnACR	3.75 ± 1.91	3.76 ± 1.97	0.91	3.72 ± 1.90	3.77 ± 1.95	0.003
Periods of the monotherapy (month)	31.8 ± 23.1	23.9 ± 14.0	<0.001	25.1 ± 18.3	24.7 ± 14.5	0.03
Periods of the combination therapy (month)	38.8 ± 18.6	28.5 ± 13.5	<0.001	31.6 ± 15.0	31.9 ± 14.0	0.02
Total periods of the study (month)	70.6 ± 27.0	52.4 ± 15.7	<0.001	56.7 ± 19.4	56.6 ± 14.7	0.006
Concomitant medications						
Sulphonylurea	108 (33%)	91 (29%)	0.34*	58 (29%)	64 (32%)	0.06
Metformin	169 (51%)	190 (61%)	0.01*	115 (57%)	114 (56%)	0.01
Insulin	141 (43%)	140 (45%)	0.56*	95 (47%)	90 (44%)	0.05
Pioglitazone	35 (11%)	51 (16%)	0.03*	29 (14%)	29 (14%)	0
αGI	40 (12%)	48 (15%)	0.22*	30 (15%)	29 (14%)	0.01
Glinide	14 (4.2%)	14 (4.5%)	0.87*	11 (5%)	11 (5%)	0
RAS inhibitor	166 (50%)	160 (51%)	0.77*	108 (53%)	96 (47%)	0.12
CCB	128 (39%)	110 (35%)	0.37*	83 (41%)	83 (41%)	0
b blocker	53 (16%)	49 (16%)	0.92*	33 (16%)	33 (16%)	0
MRB	14 (4%)	12 (%)	0.81*	10 (5%)	9 (4%)	0.02
Diuretics	53 (16%)	30 (10%)	0.10*	23 (11%)	25 (12%)	0.03
Statin	160 (48%)	160 (51%)	0.46*	109 (54%)	98 (45%)	0.11

The left panel shows the unadjusted model of the full analysis set (FAS) with multiple imputation (MI). The right panel shows the PS-matching model. Values represent the mean±SD or n/total n (%). *p*-values were determined by an unpaired t test or *chi-square test. Abbreviations: αGI, alpha glucosidase inhibitor; BMI, body mass index; BW, body weight; DBP, diastolic blood pressure; CCB, calcium channel blocker; eGFR, estimated glomerular filtration; FAS, full analysis set; GLP1Ra, glucagon-like peptide 1 receptor agonists; HbA_1c_, glycated hemoglobin A_1c_; LnACR, logarithmic value of urine albumin-to-creatinine ratio; MAP, mean arterial pressure; MI, multiple imputation; MRB, mineral corticoid receptor blocker; PS, propensity score; RAS, renin-angiotensin system inhibitor; SBP, systolic blood pressure; SGLT2i, sodium-glucose cotransporter inhibitors.

### 3.2 Annual eGFR decline on FAS

Table 2A shows that, in the analysis of 643 patients, the eGFR was significantly lower by passage of time, with values (mL/min/1.73 m^2^) of 78.5 ± 27.4 at baseline, 74.0 ± 26.5 at the time of drug addition, and 70.8 ± 26.8 at the final observation (*p* < 0.001 between each period). Similar results of eGFR decline were observed in CCA set analysis. Table 2B shows the annual eGFR decline (mL/min/1.73 m^2^/year) was −2.6 ± 9.9 in monotherapy with a period of 28.0 ± 19.6 months and −1.2 ± 6.0 in combination therapy with a period of 33.8 ± 17.1 months. The difference was statistically significant (*p* = 0.005). Table 2 also shows the changes in eGFR and annual eGFR decline depending on the preceding drug in the unadjusted model. A significant difference in annual eGFR decline was observed in patients in the SGLT2i-preceding group before and after the initiation of combination therapy (*p* < 0.001).

**TABLE 2:** The renal function and eGFR decline during the study periods on unadjusted model (FAS with MI).

A) Changes in eGFR (mL/min/1.73m^2^)												
	FAS						CCA					
	At baseline	At the time of drug addition	At the final observation	*p*-value*			At baseline	At the time of drug addition	At the final observation	*p*-value*		
				Baseline vs. addition	Addition vs. final observation	Baseline vs. final observation				Baseline vs. addition	Addition vs. final observation	Baseline vs. final observation
Total (*n* = 643)	78.5 ± 27.4	74.0 ± 26.5	70.8 ± 26.8	<0.001	<0.001	<0.001	78.8 ± 28.6	74.0 ± 27.6	70.1 ± 27.8	<0.001	<0.001	<0.001
SGLT2i-preceding group (*n* = 331 in FAS *n* = 277 in CCA)	78.2 ± 26.0	73.2 ± 26.0	71.4 ± 26.1	<0.001	0.06	<0.001	79.1 ± 26.0	74.0 ± 26.4	72.2 ± 26.6	<0.001	0.22	<0.001
GLP1Ra-preceding group (*n* = 312 in FAS n = 191 in CCA)	78.8 ± 28.7	74.7 ± 27.0	70.1 ± 27.5	<0.001	0.06	<0.001	78.5 ± 30.6	73.9 ± 28.7	68.3 ± 28.9	<0.001	<0.001	<0.001

B) Annual eGFR decline (mL/min/1.73 m^2^/year)							
		FAS			CCA		
		From at baseline to at the time of drug addition	From the time of drug addition to the final observation	*p*-value^∗^	From at baseline to at the time of drug addition	From the time of drug addition to the final observation	*p*-value^∗^
Total (*n* = 643)	Treatment periods (months)	28.0 ± 19.6	33.8 ± 17.1		28.3 ± 20.3	34.3 ± 17.3	
	eGFR decline (mL/min/1.73 m^2^/year)	−2.6 ± 9.9	−1.2 ± 6.0	0.005	−2.5 ± 10.1	−1.4 ± 6.2	<0.001
	Adjusted eGFR decline (mL/min/1.73 m^2^/year)*				−2.1 ± 2.7	−1.6 ± 2.4	0.002
SGLT2i-preceding group (*n* = 331 in FAS *n* = 277 in CCA)	Treatment periods (months)	23.9 ± 14.0	28.5 ± 13.5		24.0 ± 14.6	28.7 ± 13.8	
	eGFR decline (mL/min/1.73 m^2^/year)	−3.4 ± 9.5	−0.9 ± 6.5	<0.001	−3.3 ± 9.0	−0.8 ± 6.8	0.004
	Adjusted eGFR decline (mL/min/1.73 m^2^/year)*				−2.3 ± 2.5	−1.7 ± 2.3	0.01
GLP1Ra-preceding group (*n* = 312 in FAS *n* = 191 in CCA)	Treatment periods (months)	31.8 ± 23.1	38.9 ± 23.1		31.9 ± 23.5	39.1 ± 18.6	
	eGFR decline (mL/min/1.73 m^2^/year)	−1.9 ± 10.3	−1.6 ± 5.3	0.69	−1.8 ± 11.0	−2.0 ± 5.7	0.78
	Adjusted eGFR decline (mL/min/1.73 m^2^/year)*				−2.0 ± 2.9	−1.5 ± 2.4	0.06

Changes in eGFR (A) and Annual eGFR decline (B) are shown respectively. In complete case analysis (CCA) set, values of adjusted eGFR decline were shown. Adjusted eGFR decline = 14.48 – eGFR at baseline × 0.087 + 1.504 × (if female) – 0.069 × Age + 0.052 × BW - 0.097 × BMI - 0.018 × SBP - 0.032 × DBP - 0.048 × HbA1c (mol/l) + Treatment periods(months) × 0.022. Values are mean±SD *p-values were analyzed by paired t test among two groups or by the general linear mixed model with Bonferroni correction among three groups.

### 3.3 Adjusted eGFR decline on CCA

The calculated formula is as follows; adjusted eGFR decline = 14.48 – eGFR at baseline × 0.087 + 1.504 × (if female) – 0.069 × Age + 0.052 × BW - 0.097 × BMI - 0.018 × SBP - 0.032 × DBP - 0.048 × HbA1c (mol/L) + Treatment periods(months) × 0.022.

Although this adjustment could not eliminate all the effects of confounding factors, annual eGFR decline (mL/min/1.73 m^2^/year) significantly changed from −2.1 ± 2.7 to −1.6 ± 2.4 (*p* = 0.002) among all patients. In the analysis depending on the preceding drugs, annual eGFR decline (mL/min/1.73 m^2^/year) significantly changed from −2.3 ± 2.5 to −1.7 ± 2.3 in SGLT2i-preceding group (*p* = 0.001), whereas that changed from −2.0 ± 2.9 to −1.5 ± 2.4 in GLP1Ra-preceding group (*p* = 0.06) (Table 2B).

Multiple regression analysis identified that 1) eGFR at the time of drug addition, 2) change in HbA1c from at the time of drug addition to the final observation, and 3) the preceding of SGLT2i were independent and significant determinants of eGFR decline. These regression coefficient values were −0.10 (95% CI, −0.14, −0.05, *p* < 0.001), 0.08 (95% CI, 0.01, 0.16, *p* = 0.02), and 2.68 (95% CI, 0.27, 5.01, *p* = 0.03), respectively.

### 3.4 Results of PS-matching model

Using the PS matching method, a matching model of 203 patients in each group was constructed (Supplementary Figure S2).

#### 3.4.1 eGFR decline on PS-matching model

**FIGURE 1:**
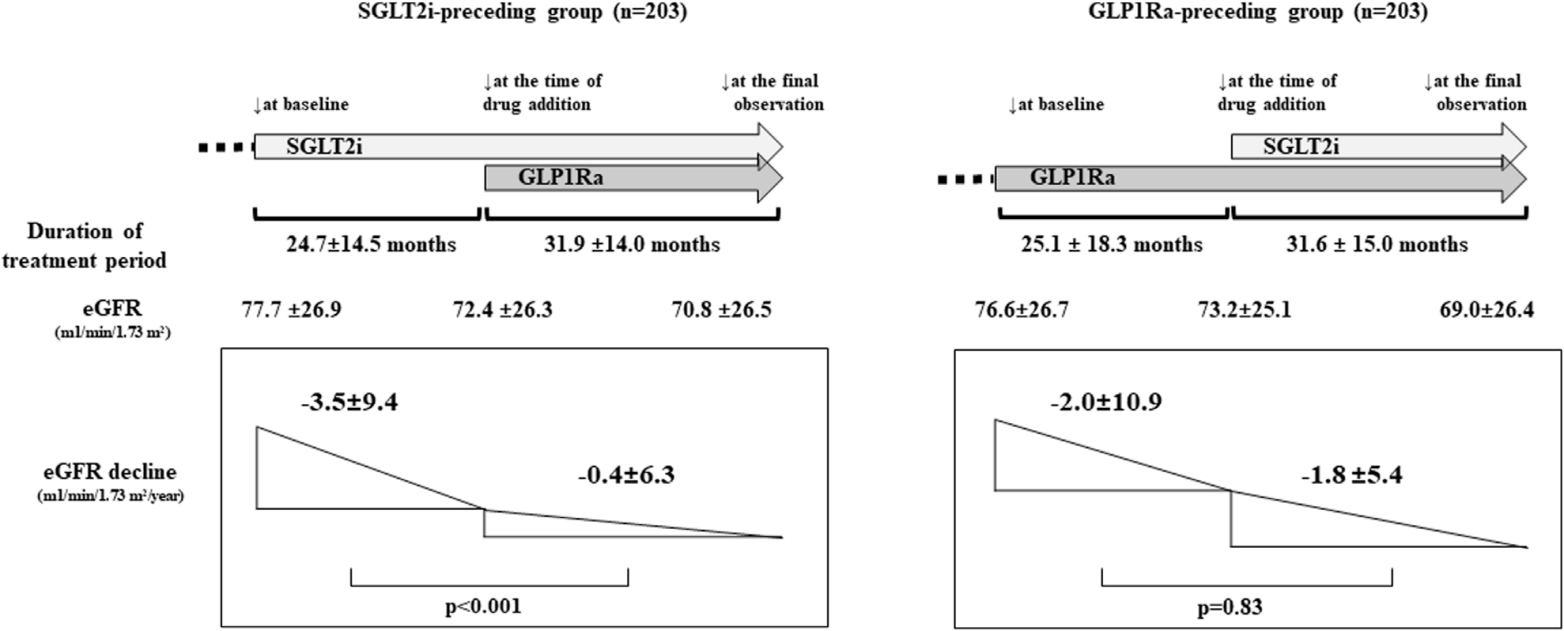
The renal function and estimated glomerular filtration rate (eGFR) decline before and after sodium-glucose cotransporter inhibitors (SGLT2i) and GLP-1 receptor agonists (GLP1Ra) combination therapy (propensity score [PS]-matching model). The eGFR at baseline, at the time of addition of drug, and the final observation were shown. (*p* < 0.001; at baseline vs. at the time of drug addition, and at baseline vs. at the final observation. *p* = 0.33; at the time of drug addition vs. the final observation in the SGLT2i-preceding group. *p* < 0.001 between each point in the GLP1Ra-preceding group. *p*-values were analyzed by general linear mixed model). The administration of SGLT2i prior to GLP1Ra significantly reduced the annual eGFR decline. *p*-values were analyzed by a paired *t*-test.

For the evaluation of the eGFR decline before and after combination therapy, we analyzed the eGFR decline separately for the SGLT2i-preceding and GLP1Ra-preceding groups in the PS-matching model. In the SGLT2i-preceding group, the annual eGFR decline (mL/min/1.73 m^2^/year) was −3.5 ± 9.4 and −0.4 ± 6.3 in the monotherapy and combination therapy periods, respectively, (*p* < 0.001). On the other hand, in the GLP1Ra-preceding group, the annual eGFR decline (ml/min/1.73 m^2^/year) was −2.0 ± 10.9 and −1.8 ± 5.4 in the monotherapy and combination therapy periods, respectively. They did not differ to a statistically significant extent (*p* = 0.83) (Figure 1).

#### 3.4.2 LnACR on PS-matching model

The LnACR values at baseline, at the time drug addition, and at the final observation were 3.77 ± 1.95, 3.79 ± 1.99, and 3.94 ± 2.00, respectively, in the SGLT2i-preceding group. Those values were 3.72 ± 1.90, 3.85 ± 1.95, and 3.78 ± 1.78, respectively, in the GLP1Ra-preceding group. None of the groups showed a significant difference in LnACR at any of the three time points.

#### 3.4.3 Changes in other clinical findings on PS-matching model

In comparison to baseline, lower HbA_1c_, BW, SBP and DBP were observed after combination therapy with SGLT2i and GLP1Ra. Table 3 shows the changes in clinical characteristics before and after combination therapy in the PS-matching model. In the monotherapy period, the HbA_1c_ value did not differ between the groups; however, lower HbA1c levels were observed after combination therapy in both the SGLT2i-preceding and GLP1Ra-preceding groups (both *p* < 0.001). A lighter BW was observed in the monotherapy period (*p* = 0.03 in the SGLT2i-preceding group and *p* = 0.007 in the GLP1Ra-preceding group), and further lighter BW was observed in both groups in the combination therapy period (both *p* < 0.001) (Table 3). There was no difference in SBP or DBP. However, in the GLP1Ra-preceding group, a further decrease in SBP was observed (*p* = 0.03).

**TABLE 3:** Clinical characteristics before and after combination therapy with SGLT2i and GLP1Ra depending on the preceding drug in the PS-matching model.

	The SGLT2i-preceding group (*n* = 203)						The GLP1R-preceding group (*n* = 203)					
	At baseline	At the time of drug addition	At the final observation	*p*-value*			At baseline	At the time of drug addition	At the final observation	*p*-value^∗^		
				Baseline vs. addition	Addition vs. final observation	Baseline vs. final observation				Baseline vs. addition	Addition vs. final observation	Baseline vs. final observation
HbA_1c_ (mol/mol [%])	71.9 ± 18.2 (8.7 ± 1.7)	69.3 ± 17.0 (8.5 ± 1.6)	62.4 ± 15.0 (7.9 ± 1.4)	0.21	<0.001	<0.001	72.8 ± 17.8 (8.8 ± 1.6)	70.9 ± 16.2 (8.6 ± 1.5)	92.9 ± 15.2 (7.9 ± 1.4)	0.46	<0.001	<0.001
BW (kg)	78.8 ± 17.0	77.8 ± 16.9	75.5 ± 17.2	0.03	<0.001	<0.001	78.7 ± 18.5	77.3 ± 18.4	73.6 ± 18.3	0.007	<0.001	<0.001
SBP (mmHg)	134.7 ± 19.4	130.4 ± 18.6	128.9 ± 17.4	0.003	1.0	<0.001	133.1 ± 19.1	132.5 ± 17.7	129.3 ± 16.1	1.0	0.03	0.01
DBP (mmHg)	78. ± 13.5	75.8 ± 12.4	74.6 ± 12.5	0.01	0.50	<0.001	76.7 ± 12.4	75.8 ± 11.8	74.6 ± 12.3	0.61	0.63	0.03

Treatment periods (months); The SGLT2i-preceding group; from baseline to the time of addition of drug (24.7±14.5), from the time of drug addition to the final observation (31.9±14.0). The GLP1Ra-preceding group; from baseline to at the time of drug addition (25.1±18.3), from the time of drug addition to the final observation (31.6±15.0). Values present the mean±SD. *p-values were analyzed between two groups by a paired t test, among three groups by a general linear mixed model with Bonferroni correction. Abbreviations: BW, body weight; SBP, systolic blood pressure; DBP, diastolic blood pressure.

## 4 Discussion

We performed this *post hoc* study to analyze the relationship between the use of SGLT2i and GLP1Ra combination therapy and renal function in T2D patients. We observed that the annual eGFR decline in the monotherapy period of SGLT2i or GLP1Ra (−2.6 ± 9.9 mL/min/1.73 m^2^/year) was significantly lower after combination therapy (−1.2 ± 6.0 mL/min/1.73 m^2^/year) (*p* = 0.005). In the PS matching model that adjusted the baseline characteristics of the two groups, in the SGLT2i-preceding group, the eGFR decline was significantly lower after the addition of GLP1Ras in comparison to SGLT2i alone (−3.5 ± 9.4 mL/min/1.73 m^2^/year to −0.4 ± 6.3 mL/min/1.73 m^2^/year, *p* < 0.001). On the other hand, the annual eGFR decline was not significantly changed by the addition of SGLT2i to the GLP1Ra-preceding group (−2.0 ± 10.9 mL/min/1.73 m^2^/year to −1.8 ± 5.4 mL/min/1.73 m^2^/year, *p* = 0.83).

In our previous study, the decrease in eGFR was slower in patients treated with SGLT2i than in those treated with GLP1Ra during monotherapy (Kobayashi et al., 2022). We hypothesize that this occurred because SGLT2i was administered for a shorter period (24.7 ± 14.5 months) in the present study, and the effect of the initial dip was greater. The mechanisms underlying this discrepancy remain unclear, but the lack of a significant difference in the annual eGFR decline in the GLP1Ra-preceding group may be due to the initial dip that occurs in the early phase of SGLT2i treatment. However, with an observation period of approximately 2 years, this effect was considered to have disappeared.

Another possible mechanism may be the difference in the mean BP at the time of drug treatment. It is well known that BP control influences the renal function decline (Soejima et al., 2022). We previously reported a lower BP in SGLT2i than in GLP1Ra (Kobayashi et al., 2019). Consistent with this observation, both SBP and DBP were significantly lower during the SGLT2i monotherapy period in the SGLT2i-preceding group. In contrast, GLP1Ra monotherapy showed no change in BP in the GLP1Ra-preceding group. The lower BP during SGLT2i monotherapy may have contributed to the subsequent reduction in annual eGFR decline with combination therapy. These findings suggest that reducing BP before the initiation of combination therapy may be related to the efficient induction of renoprotection.

In this study, no significant changes were observed in the ACR between the two groups. Considering these results, the degree of ACR was relatively low; therefore, it was difficult to determine the effect of combination therapy.

A series of large-scale randomized controlled trials (RCTs) demonstrated that SGLT2i use reduced the risk of kidney disease progression in patients with and without diabetes (Nuffield Department of Population Health Renal Studies and Consortium, 2022). The beneficial effects of SGLT2i on albuminuria and eGFR decline in DKD have been demonstrated by EMPA-REG OUTCOME (Wanner et al., 2016), CANVAS (Perkovic et al., 2018), DECLARE-TIMI58 (Mosenzon et al., 2019), and CREDENCE (Perkovic et al., 2019). These effects were consistent in DAPA-CKD (Heerspink et al., 2020; Heerspink et al., 2021) and EMPA-KIDNEY (The EMPA-KIDNEY Collaborative Group et al., 2023), which included CKD patients with and without diabetes. Recently, a *post hoc* analysis of SUSTAIN6 and PIONEER6 demonstrated that semaglutide slowed the eGFR decline in T2D patients with a high risk of CVD (Tuttle et al., 2023), suggesting that GLP1Ra has potentially favorable effects on the renal function.

With combination therapy of SGLT2i and GLP1Ra, the preceding drug did not affect the renal outcome in the main analysis of RECAP study (Kobayashi et al., 2023). In this *post hoc* analysis, the SGLT2i-preceding group had a slower rate of eGFR decline in comparison to the GLP1Ra-preceding group, and multiple regression analysis identified the preceding of SGLT2i as an independent and significant factor of change in eGFR decline. Interestingly, we also demonstrated a combination of both drugs had additive renoprotection. Furthermore, the addition of GLP1Ra to SGLT2i showed an advantage in the decrease in BW. These findings suggest that there is no problem in starting SGLT2i first with the expectation of renoprotection, however, if a stronger protective effect is desired, a combination of GLP1Ra is preferable. In addition, GLP1Ra may be used first in patients with obesity who should prioritize BW reduction. Recent studies demonstrated that SGLT2i had cardioprotective effects in HFpEF (heart failure with preserved ejection fraction) and HFrEF (heart failure with reduced ejection fraction) with or without diabetes (Solomon et al., 2022; Cortes et al., 2023). On the other hand, GLP1Ra can be prescribed only for patients with T2D. Therefore, it remains unclear whether similar results can be observed in patients without T2D.

## 5 Limitations

The present study was associated with several limitations. First, the data were analyzed as a *post hoc* analysis from a retrospective study and may have been insufficient to evaluate eGFR decline. Second, the relatively low numbers of participants may have resulted in insufficient power to detect differences in the effect of treatment on eGFR decline. Third, it remains unclear whether our results can be generalized to T2D patients. The analysis target was patients who required combination therapy with SGLT2i and GLP1Ra for the treatment of T2D. Combination therapy has been used for glycemic management and/or BW reduction but not for renoprotection. Indeed, obese patients were enrolled (mean BMI: 29.2 ± 5.3 kg/m^2^ in the SGLT2i-preceding group and 29.4 ± 5.5 kg/m^2^ in the GLP1Ra-preceding group) in this study, and the influence of combination therapy on obesity-related glomerulopathy was not evaluated sufficiently. Fourth, we could not exclude the possibility that clinicians selected SGLT2i first because of renoprotective properties of SGLT2i. Supplementary Figure S3 shows the distribution of the years at the initiation of treatment depending on the drug. Approximately one-third of patients in GLP1Ra preceding group started GLP1Ra before 2014 when SGLT2i were not commercially available in Japan. In contrast, some patients in the SGLT2i-preceding group may have started SGLT2i based on expectation of renoprotective effects. It is possible that patients at high risk for renal outcomes started SGLT2i first, which may have influenced the eGFR decline in this study. Fifth, the population included in this study was only Japanese. Therefore, the generalizability of these results to other races requires further validation. Finally, the PS-matching could balance known confounding variables as much as possible, including gender, duration of diabetes, HbA1c, BMI and BP to eliminate baseline differences between groups, but it could not ensure that all measured baseline characteristics were matched and consider the influence of unknown variables.

## 6 Conclusion

In conclusion, this retrospective study demonstrated that SGLT2i and GLP1Ra combination therapy could be beneficial in reducing the annual eGFR decline in patients with T2D. Among the combination therapies, the SGLT2i-preceding group showed a significant reduction in annual eGFR decline, while the GLP1Ra-preceding group did not.

Further studies are required to clarify the additive effect of SGLT2i and GLP1Ra combination therapy on renal protection. In particular, the effects of preceding drugs on the rate of annual eGFR decline should be investigated in a prospective RCT.

## Data Availability

The original contributions presented in the study are included in the article/Supplementary Material, further inquiries can be directed to the corresponding author.

## Supplementary material

SUPPLEMENTARY FIGURE S1 Patients with T2D who were treated with both SGLT2i and GLP1Ra from April 2010 to December 2021. Patients who had been on monotherapy for at least 6 months and on combination therapy for at least 12 months were registered in the study.

SUPPLEMENTARY FIGURE S2 A total of 688 patients were registered, and 45 patients were excluded. The data of 643 patients (SGLT2i-preceding group, *n* = 312; GLP1Ra-preceding group, *n* = 331) were analyzed as the FAS. The multiple imputation method was applied to FAS data. Propensity score matching was conducted to compare the annual ΔeGFR and LnACR.

SUPPLEMENTARY FIGURE S3 Distribution of years at the initiation of treatment for each type of drug. Approximately one-third of patients in GLP1Ra preceding group started GLP1Ra before 2014 when SGLT2i were not commercially available in Japan. The vertical axis shows years and the horizontal axis shows numbers of patients.
