# Supplementary figures and images for: Influence of the combination of SGLT2 inhibitors and GLP-1 receptor agonists on eGFR decline in type 2 diabetes: post-hoc analysis of RECAP study
Source: Front Pharmacol. 2024 Mar 27;15:1358573. doi: 10.3389/fphar.2024.1358573 (PMC11005912; doi:10.3389/fphar.2024.1358573)

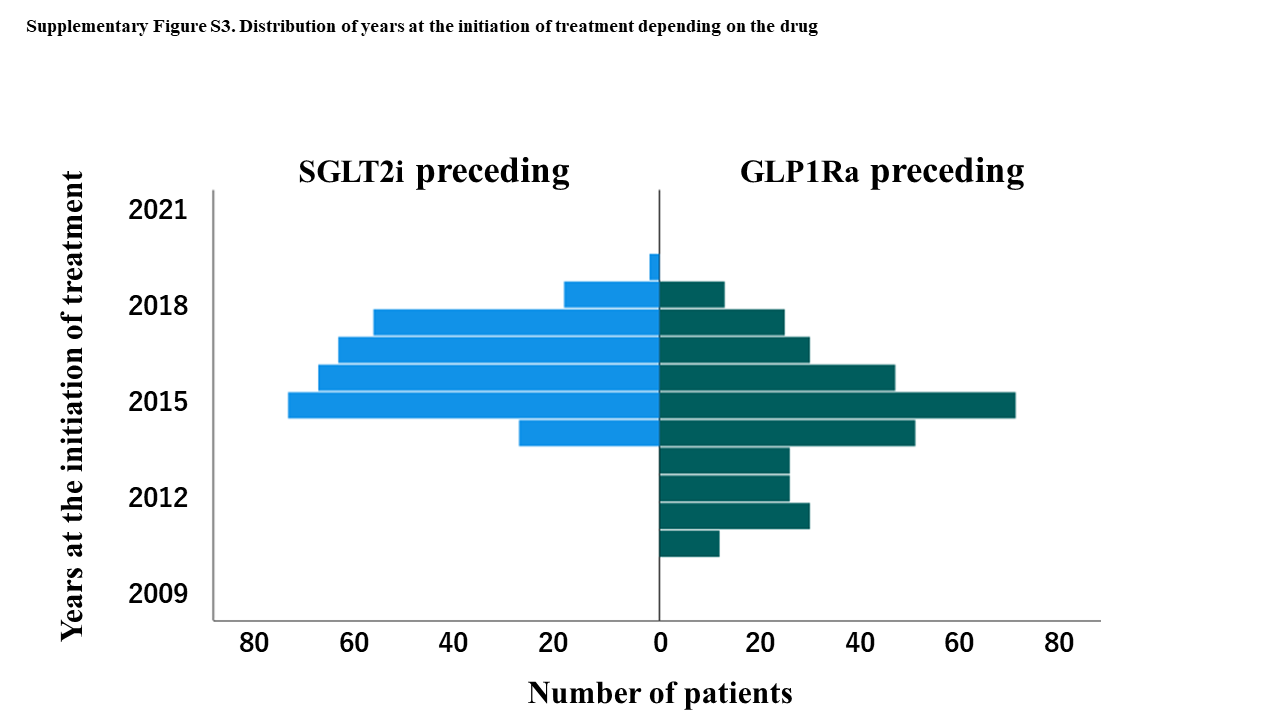

Supplement: Supplementary file 1 [file Figure4.TIF]

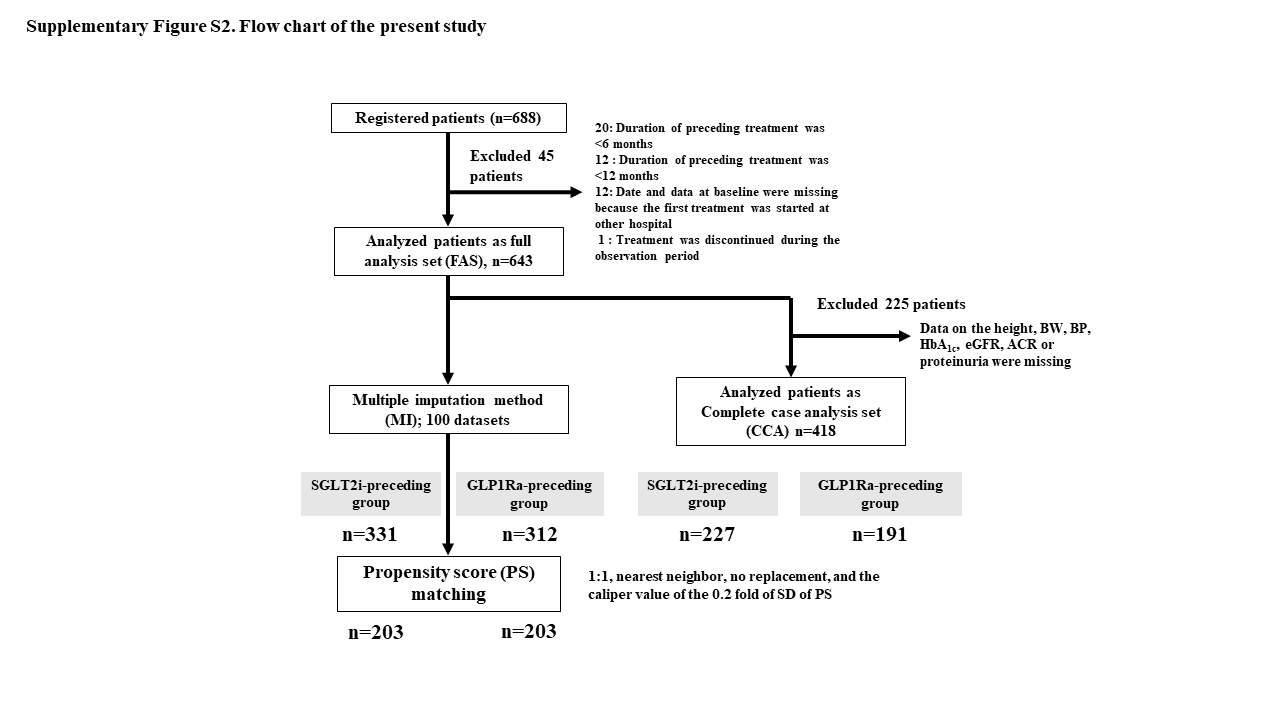

Supplement: Supplementary file 2 [file Figure3.TIF]

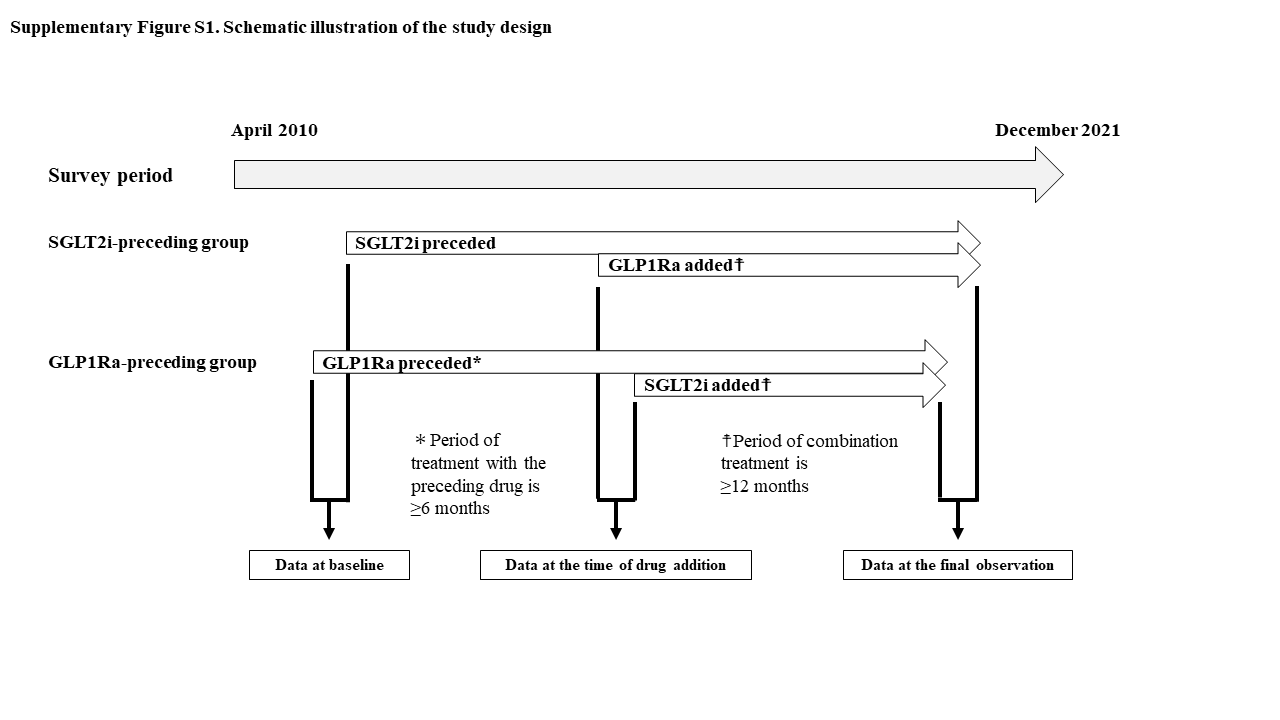

Supplement: Supplementary file 3 [file Figure2.TIF]
